# From TTP to Glomerulonephritis: A Lifetime of Lupus

**DOI:** 10.1155/2021/6654748

**Published:** 2021-01-04

**Authors:** Fadi Kharouf, Sigal Shahar, Yoav Hershkovitz, Alaa Shaheen, Areej Bayatra, Asa Kessler, Yuval Ishay

**Affiliations:** ^1^Department of Medicine, Hebrew University-Hadassah Medical Center, Jerusalem, Israel; ^2^Rheumatology Unit, Hebrew University-Hadassah Medical Center, Jerusalem, Israel; ^3^Gastroenterology Unit, Hebrew University-Hadassah Medical Center, Jerusalem, Israel

## Abstract

We report the case of a 56-year-old male patient, who over two decades, sequentially presented with a combination of clinical manifestations. These included thrombotic thrombocytopenic purpura (TTP), right leg deep vein thrombosis (DVT), and eventually constitutional symptoms, arthralgia, diffuse lymphadenopathy, pancytopenia, skin rash, pericarditis, and glomerulonephritis. Serologic tests and renal pathology uncovered a diagnosis of systemic lupus erythematosus (SLE), and immunosuppressive therapy was initiated. Soon after, the patient developed striking cytomegalovirus (CMV) viremia, requiring prolonged antiviral therapy and reduction of immunosuppression. Finally, an acute embolic stroke complicated the disease course. Prompt interventions allowed an excellent clinical outcome.

## 1. Introduction

Systemic lupus erythematosus (SLE) is the prototypical systemic autoimmune disease, characterized by a wide spectrum of clinical manifestations, often exhibiting a relapsing-remitting course [1]. As treatment is based on immunosuppressive therapy, infections pose a major iatrogenic complication [2]. Here, we report a unique case of a middle-aged male, gradually presenting with a diverse constellation of systemic manifestations over a prolonged period. We discuss the clinical presentation, diagnostic implications, therapeutic decisions, and treatment complications.

## 2. Case Presentation

A 56-year-old male presented to the emergency department with a 2-month history of extreme weakness, fatigue, and anorexia. His past medical record was remarkable for a previous event of thrombotic thrombocytopenic purpura (TTP) 20 years earlier. He was then treated with plasmapheresis, aspirin, and corticosteroids, with complete recovery. 18 months thereafter, he was diagnosed with an unprovoked right leg deep vein thrombosis (DVT), for which no etiology was found. Noticeably, antiphospholipid antibodies (APLAs) were negative.

Similar constitutional symptoms had plagued the patient before. Several years prior to his current presentation, he was admitted for weakness and weight loss (30 kg over a few months). He then underwent a thorough investigation, including blood tests, whole-body computed tomography (CT), and positron emission tomography/CT (PET/CT). Diffuse hypermetabolic lymphadenopathy, involving axillary, abdominal, and retroperitoneal lymph nodes (LNs), was seen. Excisional biopsy of a pelvic LN (PN) and bone marrow (BM) biopsy were performed, both being unremarkable. Immune serologies sent at that time showed only a weakly positive antinuclear antibody (ANA). The differential diagnosis comprised a systemic infection, an indolent neoplastic process, and autoimmune lymphoproliferative syndrome (ALPS). None of these entities was proven however, leaving the patient undiagnosed despite regular follow-up. Thereafter, he spontaneously recovered, enjoying several uneventful years.

**Figure 1:**
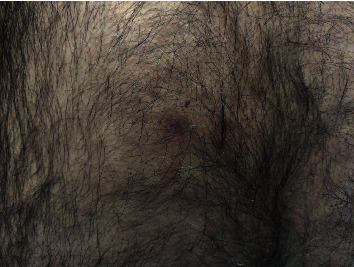
Lichen planus-like lesions on the patient's back. On biopsy, the positive lupus band test was found, signifying immune complex deposition.

Upon the patient's current admission, physical examination revealed mild bilateral leg edema and numerous lichen-like skin lesions (see Figure 1). Recurrent fever spikes of up to 39°C were recorded, with otherwise normal vital signs. Blood tests were extremely abnormal, showing pancytopenia, hypoalbuminemia, high creatinine levels, and elevated inflammatory markers (see Table 1). While whole-body CT displayed no noteworthy findings, PET/CT demonstrated widespread hypermetabolic lymphadenopathy and diffusely increased BM uptake. BM and groin LN biopsies were performed, again being noncontributory. Peripheral blood flow cytometry, however, showed a high proportion (7%) of double-negative (DN) T cells (negative for both CD4^+^ and CD8^+^). Blood cultures and broad bacterial and viral serologies were negative. The patient's urinalysis was significant for hematuria and proteinuria, and urine microscopy revealed red blood cell (RBC) casts, along with dysmorphic RBCs. 24-hour urine collection demonstrated nephrotic range proteinuria (3.9 g/d).

During this time, the patient's clinical condition was one of the ongoing weakness, fatigue, and dyspnea. He also suffered from pleuritic chest pain and progressive, asymmetric leg edema. Further work-up directed at these complaints showed an extensive right leg DVT, elevated cardiac enzymes, and new-onset atrial fibrillation (AF). In addition, a small pericardial effusion was found on transesophageal echocardiography (TTE). Therapy with enoxaparin was started for his DVT and AF, and broad-spectrum antibiotic coverage was instituted for a suspected bacterial infection. Pericarditis was added to this ever-growing list of diagnoses.

Results of ANA and anti-double-stranded deoxyribonucleic acid (anti-dsDNA) antibody were reported at this stage, both being strongly positive, along with low complement levels (see Table 2). Notably, APLAs were negative. In light of the patient's deteriorating renal function and active urinary sediment, kidney biopsy was performed. Lupus nephritis stages 3 and 5 (focal proliferative and membranous glomerulonephritis, respectively) were present on pathology. Biopsy of the skin lesions demonstrated immune complex (lupus band) deposition and a lichen planus-like pathological picture. The patient was thus diagnosed with SLE and started on high-dose corticosteroid therapy, hydroxychloroquine, mycophenolate mofetil, and ramipril, with impressive clinical and laboratory improvement. After a 4-week hospital stay, he was eventually discharged for follow-up at the rheumatology and nephrology outpatient clinics.

Less than one month after discharge, the patient was readmitted due to a painful right calf ulcer, productive cough, and general weakness. Thorough work-up revealed significant cytomegalovirus (CMV) viremia (>925,000 copies/mL), with no signs of visceral involvement upon bronchoscopy, sigmoidoscopy, and ophthalmologic evaluation. The leg ulcer was attributed to postphlebitic syndrome, and biopsy of the ulcer was noncontributory. Cultures from both the ulcer and bronchoalveolar lavage fluid grew *Pseudomonas aeroguinosa*, and therapy with ciprofloxacin was started. Besides, mycophenolate mofetil and prednisone doses were reduced in an attempt to minimize immunosuppression, and intravenous ganciclovir therapy was initiated. The lack of clear signs of end-organ involvement has made it difficult to define the required duration of ganciclovir treatment, and the CMV viremia was monitored instead. Following the gradual elimination of viremia, antiviral therapy was stopped. Diligent wound care, compression stockings, and antibiotic treatment allowed stepwise healing of the ulcer.

Three months later, the patient was again admitted for sudden-onset dysarthria and right-sided hemiparesis. Brain CT angiography showed left middle cerebral artery branch obstruction, consistent with an embolic etiology. An emergent thrombectomy was performed. TTE, transesophageal echocardiography (TEE), and carotid Doppler studies revealed no embolic source. Aspirin and high-dose statin were added, and the patient continued apixaban. Gradually, he was able to regain excellent functional capacity.

As of today, one year after his index presentation, the patient is well and suffers no residual neurologic deficits. His most recent labs, including blood counts, chemistry panels, complement levels, and urinalysis, are unremarkable. His proteinuria has declined to 170 mg/d, and CMV titers in blood are undetectable.

## 3. Discussion

Our patient was eventually diagnosed with SLE. Over 20 years, he presented with an assembly of disease manifestations, including hematologic, musculoskeletal, dermatologic, cardiac, and renal phenomena. Initially experiencing events of TTP and DVT, he went on to develop, over many years and in a relapsing-remitting manner, constitutional symptoms, arthralgia, lymphadenopathy, pancytopenia, skin lesions, pericarditis, and glomerulonephritis. Past immune serologies showed only a weakly positive ANA, with normal complement levels. The absence of classic lupus manifestations in the past obscured the diagnosis, and it was not until significant visceral involvement occurred many years thereafter, that the correct conclusions were drawn. In these days of extensive testing and follow-up, “the great pretender,” lupus, appears to present a lesser diagnostic dilemma than in the past. Atypical manifestations, combined with spontaneous disease remissions, may nonetheless obfuscate the clinical picture.

Constitutional symptoms, including fatigue, anorexia, fever, and weight loss, are commonly seen in patients with SLE. Fatigue is a frequent, sometimes disabling, disease symptom. Its etiology is multifactorial and includes chronic inflammation, cytokine dysregulation, side effect of medications, comorbidities, and hormonal imbalances [3]. Fever occurs in 42–86% of SLE patients [4]. Its work-up can be challenging, and the disease occasionally presents as fever of unknown origin (FUO) [5].

SLE is known to have myriad hematologic manifestations, many of which appeared in our case. Lymphadenopathy is a common, often unappreciated feature. LNs are usually soft and nontender and may fluctuate in size with disease exacerbations. Biopsy usually shows reactive hyperplasia [6]. 50% of patients with SLE develop anemia, usually due to chronic inflammation and iron deficiency, and less often due to autoimmune hemolysis [7]. White blood cell abnormalities are also common in lupus and include leukopenia, lymphopenia, and neutropenia [8]. Thrombocytopenia, when related to active disease, tends to be lower than 50,000/mm^3^ and usually necessitates aggressive therapy [9]. While TTP may be a rare manifestation of SLE, it is seldom the presenting feature [10]. Thrombosis, on the contrary, is a well-known finding in the disease and is multifactorial. Despite APLAs being the most important risk factor, 40% of SLE patients negative for these antibodies will still experience a thrombotic event. Other factors that contribute to thrombosis in lupus include inflammatory disease activity, medications, and traditional risk factors (including smoking, diabetes, and hypertension) [11]. Immunologically, it is noteworthy that SLE patients may have increased numbers of DN T cells in peripheral blood, probably driven by excessive T cell stimulation. These cells synthesize interleukins and can stimulate B cells, contributing to the pathogenesis of kidney damage, for instance [12].

Regarding cardiac involvement, SLE can affect any layer of the heart. Pericardial effusion secondary to pericarditis is the most common manifestation [13]. Valvular heart disease is also well known in SLE. Left-sided valvular thickening is the predominant finding, followed by valvular vegetations, regurgitation, and stenosis [14]. It is worth mentioning that, in Libman–Sacks endocarditis complicating SLE, the sterile valvular vegetations have a greater tendency to embolize than in infective endocarditis, thus predisposing to neuropsychiatric disease, including ischemic stroke [15]. The latter is twice as common in SLE patients as compared to the general population. Contributing factors include embolic events, comorbidities (such as antiphospholipid syndrome and hypertension), systemic inflammation, and treatment complications [16]. AF, present in our case, is also known to be more common in SLE patients and is associated with increased mortality [17].

Skin disease is a well-characterized feature of lupus. Chronic cutaneous lupus includes a lupus-lichen overlap entity [18]. In the majority of patients with systemic disease, immunofluorescence reveals deposits at the dermal-epidermal junction in normal non-sun-exposed skin and at sites of cutaneous lesions, the so-called lupus band test [19].

Renal involvement remains one of the most devastating manifestations of SLE, with increased morbidity and mortality. Despite advances in management, 10% of the patients still progress to renal insufficiency and end-stage renal disease [20].

Complications of immunosuppression are also represented in our case. CMV viremia dominated the clinical picture, necessitating reduction of immunosuppression and institution of prolonged intravenous antiviral therapy. The heavy immunosuppressive regimens used in systemic autoimmune diseases are known to predispose to opportunistic infections. These remain the most frequent cause of hospitalization, morbidity, and mortality in SLE patients. More than half of the infections are viral, the most common being herpes zoster [2]. No guidelines in the field of rheumatology advise in favor of prophylactic therapy against CMV upon heavy immunosuppression, as opposed to the recommendations in patients undergoing BM transplantation. In cases of symptomatic CMV disease, treatment involves both the minimization of immunosuppression and the institution of antiviral therapy, typically with oral valganciclovir or intravenous ganciclovir [21]. Duration of intravenous therapy is usually guided by the target organ, lacking in our patient.

**Figure 2:**
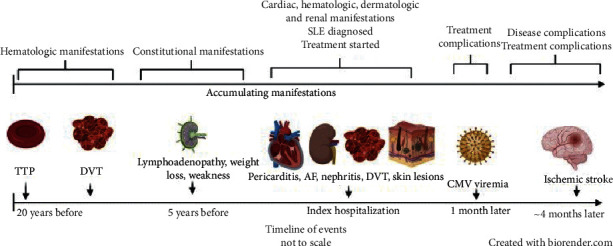
Timeline of events in the patient's course over 20 years. AF: atrial fibrillation; CMV: cytomegalovirus; DVT: deep vein thrombosis, TTP: thrombotic thrombocytopenic purpura.

We believe our case is educational in several aspects. First, the patient showed a remarkable mixture of systemic manifestations (see Figure 2), varying in severity, pattern, and timing, with the eventual diagnosis being made two decades after his initial presentation. Secondly, despite appropriate therapy, he went on to develop complications of the disease and its treatment, including DVT, AF, CMV viremia, and stroke. Finally, the correct utilization of the therapeutic armamentarium and the proper implementation of urgent interventions yielded an excellent outcome.

## Figures and Tables

**Table 1:** The patient's blood chemistry and counts upon admission.

Lab parameter	Value
Creatinine (*μ*mol/L) (62–115)	180
Albumin (g/L) (32–48)	19
Leukocytes (*μ*L) (3,790–10,330)	1,100
Hemoglobin (g/dL) (13.9–17.7)	7.5
Platelets (*μ*L) (166,000–389,000)	62,000
Neutrophils (*μ*L) (1780–7,000)	500
Lymphocytes (*μ*L) (1,070–3,120)	300
ESR (mm/hr) (1–20)	62
CRP (mg/dL) (0–0.5)	2.7
Haptoglobin (mg/dL) (3–200)	296

Normal range values appear in brackets. ESR: erythrocyte sedimentation rate; CRP: C-reactive protein.

**Table 2:** The patient's immune serologies, completed during his admission.

Lab parameter	Value
ANA (0/4)	4/4
Anti-DNA (U/mL) (<25)	>200
C3 (U/mL) (90–180)	35
C4 (mg/dL) (10–40)	4
Coombs test	Positive

Normal range values appear in brackets. ANA: antinuclear antibodies; Anti-DNA: anti-deoxyribonucleic acid; Anti-dsDNA: anti-double-stranded deoxyribonucleic acid; C: complement.

## Data Availability

All data used to support our findings are included within the article, and any additional data will be provided by the corresponding author upon reasonable request.

## Abbreviations

AF: Atrial fibrillation
ALPS: Autoimmune lymphoproliferative syndrome
ANA: Antinuclear antibody
Anti-dsDNA: Anti-double-stranded deoxyribonucleic acid
BM: Bone marrow
C: Complement
CMV: Cytomegalovirus
CT: Computed tomography
CRP: C-reactive protein
DN: Double negative
DVT: Deep vein thrombosis
RBC: Red blood cell
ESR: Erythrocyte sedimentation rate
FUO: Fever of unknown origin
LN: Lymph node
PET: Positron emission tomography
SLE: Systemic lupus erythematous
TEE: Transesophageal echocardiography
TTE: Transthoracic echocardiography
TTP: Thrombotic thrombocytopenic purpura.
